# Correction: MicroRNA-101 Targets CXCL12-Mediated Akt and Snail Signaling Pathways to Inhibit Cellular Proliferation and Invasion in Papillary Thyroid Carcinoma

**DOI:** 10.32604/or.2025.064363

**Published:** 2025-06-26

**Authors:** FANG CHEN, DONGQIANG YANG, YUHUA RU, SHAN CAO, AISHE GAO

**Affiliations:** 1 Department of Pathophysiology, Henan University of Traditional Chinese Medicine, Zhengzhou, 450046, China; 2 Department of Infectious Diseases, Henan Provincial People’s Hospital, Zhengzhou, 450003, China; 3 Department of Medical Academy, Soochow University, Suzhou, 215021, China

In the article “MicroRNA-101 Targets CXCL12-Mediated Akt and Snail Signaling Pathways to Inhibit Cellular Proliferation and Invasion in Papillary Thyroid Carcinoma” (*Oncology Research*. 2019 Jun 21;27(6):691–701, doi:10.3727/096504018X15426763753594), the IHC images for CXCL12 and Bcl-2 expressions in adjacent noncancer tissues (NCT) shown in Fig. 5E were unintentionally duplicated. And Fig. 5A,B was also unintentionally duplicated. These needed corrections to ensure the accuracy and integrity of the data presented.

**Figure 5:**
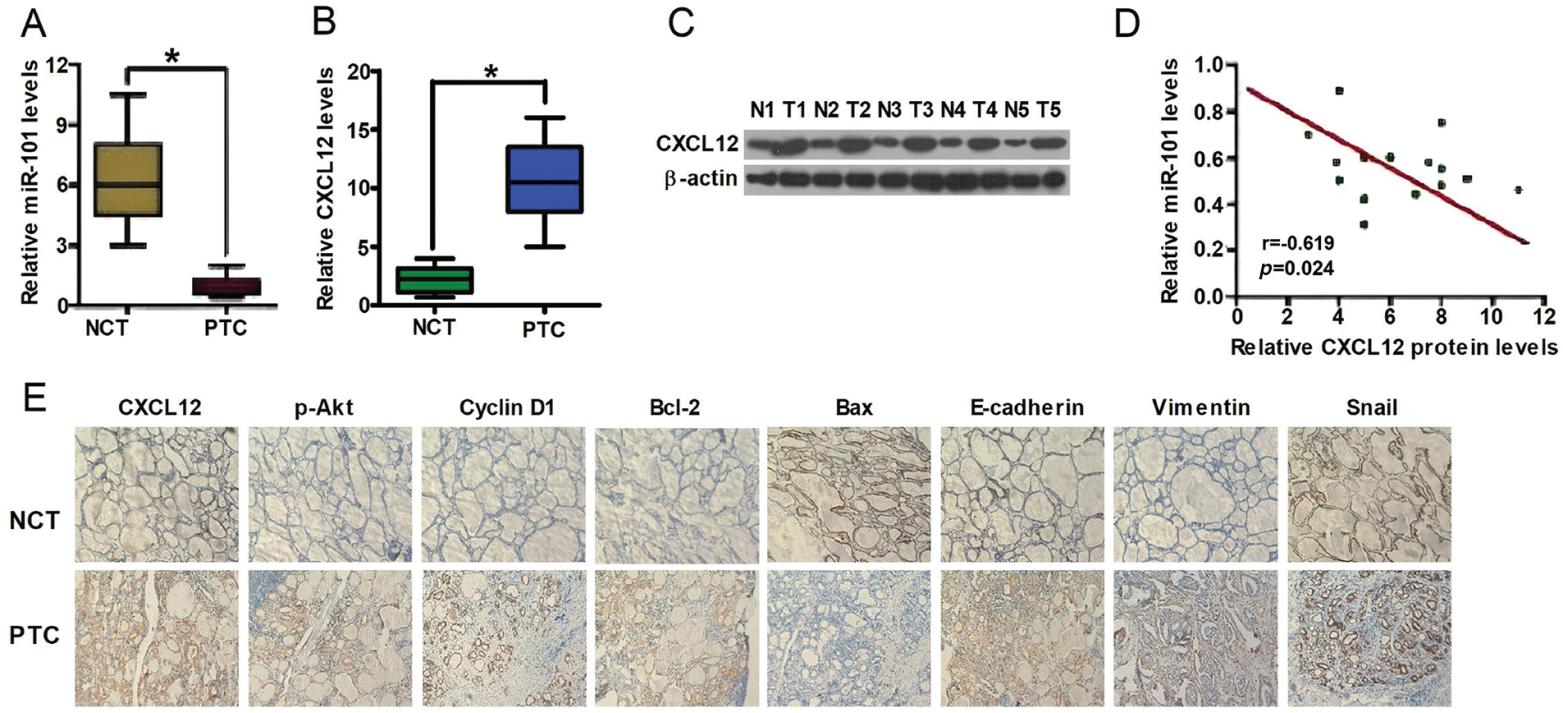
miR-101 and CXCL12 expressions in PTC tissues. (A) qPCR analyses of miR-101 expression and (B) mRNA level of CXCL12 in PTC tissues (n = 20) and in NCT tissues (n = 20). U6 and GAPDH were used as the internal controls, respectively. (C) Representative Western blot results of CXCL12 in five pairs of PTC and NCT tissues. β-Actin was used as endogenous control. (D) The correlation between miR-101 expression and CXCL12 mRNA level is shown. (E) IHC results of CXCL12, p-Akt (Ser473), cyclin D1, Bax, Bcl-2, E-cadherin, vimentin, and Snail in PTC and NCT tissues. Data are expressed as means ± SD of three separate experiments. *p < 0.05 compared with adjacent NCT. NCT, noncancer tissues.

Issue with Fig. 5A and B:

- Original Issue: Duing to an oversight, the image for Fig. 5A was mistakenly recognized as the image for Fig. 5B and incorrectly placed. This resulted in a mismatch between the image and the actual experimental conditions it was intended to depict.
- Reason for Change: To accurately represent the qPCR results of CXCL12 mRNA from various tissues, we have revised Fig. 5B. The new image correctly labels the relative CXCL12 mRNA levels and accurately corresponds to the intended experimental setup.
- Impact on Results: The revision of Fig. 5B does not involve any alteration of the legends or text associated with the Figure. It replaces the erroneous image with the correct one, ensuring that the visual data accurately corresponds to the reported experimental findings. This correction does not impact the scientific conclusions drawn in the study.

Issue with Fig. 5E:

- Original Issue: Duing to an oversight, the IHC images for CXCL12 and Bcl-2 expressions in adjacent noncancer tissues (NCT) shown in Fig. 5E were unintentionally duplicated. This resulted in a mismatch between the image and the actual experimental conditions it was intended to depict.
- Reason for Change: To accurately represent the IHC results of CXCL12 and Bcl-2 expressions from various tissues, we have revised Fig. 5E. The new image correctly labels CXCL12 and Bcl-2 and accurately corresponds to the intended experimental setup.
- Impact on Results: The revision of Fig. 5E does not involve any alteration of the legends or text associated with the Figure. It replaces the erroneous image with the correct one, ensuring that the visual data accurately corresponds to the reported experimental findings. This correction does not impact the scientific conclusions drawn in the study.

The corrected version of Fig. 5 is provided. The changes were necessary to maintain the integrity of the published work and to provide accurate visual data to support the study’s findings. The authors confirm that these corrections do not alter any of the study’s results or conclusions. This correction has been approved by the Editors-in-Chief and the Editorial Office of *Oncology Research*, and the original publication has been updated accordingly.

The authors would like to correct the figure as follows:

**Table:** 

Page No.	Exact figure to be corrected	Correction
698	Fig. 5	Replace with new Fig. 5

